# Dr. Vaughan, on Arsenic in Hydrophobia

**Published:** 1805-07-01

**Authors:** Walter Vaughan

**Affiliations:** Rochester


### *To the Editors of the Medical and Physical Journal.*

GENTLEMEN,

IN your Journal of last April is contained a letter, dated Chatham, and subscribed Thomas Hope, the object of which is to recommend the internal use of arsenic for the cure (or, perhaps, for only the prevention) of rabies canina. As I am one of those, who deem arsenic, under any administration, in the present state of medical science, a most a most formidable remedy; and as I am seriously alarmed, lest the letter in question should tend, among such as have not duly attended to the appropriate studies of their profession, to raise an unmerited reputation for one of the most deadly and unmanageable of poisons; it is my earnest request that you will insert the following remarks in your next Journal.

1. Mr. Hope asserts, that Dr. Bardsley's communication "can but excite the rising wish that some remedy may be discovered that will remove so dreadful a disorder as that of rabies canina." It seems, therefore, as if he takes it for granted that the wish to cure canine madness is as yet but a rising, or, as I understand it, a nascent wish; whereas, Gentlemen, this very wish, as you know well, had actually risen, and had become universal, even before the reign of Claudius, (Celsus, de Med. Lib. iv. Cap. 27.) Besides, the phrase, "to excite a rising wish," is so very uncommon, that I must dwell a little upon it. When we hear or read of a tumult having been excited, it suggests to us, that no tumult had pre-existed, or that there had been no rising tumult. Perhaps for "excite" we should read *incite*.

On so important an occasion as the present, (Rerum volo esse solicitudinem,) I would willingly forbear to criticise the grammar, or logic, or rhetoric of any gentleman; and, for this profession, I expect that even Mr. Hope will give me credit, when he shall again have examined the rhetorical exornation, with which his letter begins.

2. But to proceed. Dr. Bardsley having witnessed some cases of hydrophobia, that ended fatally (as, I believe, every case of it has ended,) led by experience of the efficacy of tonics, says, "if it should ever fall to my lot to treat a third case of so distressing and fatal a malady, I shall be tempted to try safe yet active doses of the arsenical solution, &c." and Mr. Hope says emphatically, "I have, to encourage the practice, (i.e. I presume, the practice which Dr. Bardsley intends to adopt) sent you the following recipe; which I have for some time had in my possession. The following is a literal transcripton, and is entitled,

"*A Receipt for the Bite of a mad Dog.*

"Take the leaves of rue, pick them from the stalk, bruise them; six ounces of garlic, picked from the stalk, and bruise them likewise; Venice treacle or mithridate and the scrapings of pewter, of each four ounces. Boil all the above ingredients over a slow fire, in two quarts of strong ale, ale, till one pint be consumed; then keep it in a bottle close stopped up, and give thereof to a man or woman, (warm it first) nine spoons full, seven mornings fasting together. To a dog six spoons full.

"This the author believes, will not, by God's blessing, fail, if it be given within nine days after the biting of a dog. Apply some of the ingredients from which the liquor was strained to the bitten place.

"This recipe was taken out of Colthorpe Church, in Lincolnshire, in 1766, the whole town being bitten by a mad dog; all that took this medicine did well, the rest that did not take it, died."—Here, Gentlemen, I must digress so far as to declare my regret, that Mr. Hope does not inform you how he came by this recipe. For to me nothing seems clearer, than that it is a mutilated and corrupt copy of Sir Theodore Mayerne's receipt, published many years ago in the Philosophical Transactions; where it stands thus: "Take leaves of rue picked from the stalks, and bruised, six ounces; of London treacle, (or, which is better, Venice treacle) garlic peeled and bruised, and fine filings of tin, each four ounces; put them into two quarts of Canary or good white wine; or in case of a nice constitution, into the same quantity of strong and well-worked ale, in an earthen vessel well stopped. Then let there be made a digestion or gentle boiling thereof, in a bath heat, for some hours, shutting in the steam. Then press it and strain it.

"The dose is from two to three ounces or more, to be taken every morning for nine days."

3. To return from my digression. In what manner is the practice of encountering hydrophobia with arsenic "encouraged," by hydrophobia having been prevented by the exhibition of *arsenic* (granting that it have been so prevented)? The means of cure, in this instance, are not at all likely to be the means of prevention likewise; for mercury, every one knows, cures syphilis, but does not prevent it; Peruvian bark cures intermittents, but does not prevent the disposition to them, when once formed, from running into action, &c. How, then, does Mr. Hope encourage that practice, which Dr. Bardsley intends to adopt?

*a.* Another question, suggested by Mr. Hope's communication, "the whole town of Colthorpe being bitten by a mad dog," (doubtless an hyperbolical auxesis,) is, What proof is there that those who took the pewter medicine would have had a timor aquæ, if they had not taken it?

it? It is a fact generally admitted, that out of twenty bitten by a dog really mad, nineteen, upon an average, escape without hydrophobia.

Mr. Hope's "literal transcription" may by some fastidious readers be thought to evince, that not the dog, but the people of Colthorpe were mad; but I will only suppose, for the present, that the dog was wonderously empowered to bite, and the people miraculously disposed to be bitten.

*b.* And another question is, If the Colthorpe remedy have ever prevented hydrophobia, (of which, however, there is not the shadow of a proof) is the use of arsenic, in any degree, authorised by it? Mr. Hope thinks it is; he says, "the chief ingredient is most probably the pewter, and that from the arsenic which enters its composition." But arsenic cannot be said to enter into the composition of pewter, because tin, which is a necessary component part of pewter, may, accidentally, be contaminated with arsenic. "Now and then," says Gren, "tin is contaminated with arsenic, but all sorts of tin are not contaminated with that metal." Mr. Hope's opinion, therefore, contains a petitio principii.

Again, although the fumes of pewter impregnated with arsenic are noxious, yet pewter is not noxious; neither is tin noxious in its metallic state; for Alston and others used to prescribe tin in very large doses, even the dose of an ounce, as an anthelmintic.

Lastly, supposing the tin to contain $\frac{1}{1152}$ part of arsenic; (and Gmelin assures us the commonest sorts of tin do not contain more) and supposing pewter and tin to be soluble (which they are not) in water, in ale, in Canary, in saliva, or in gastric liquor; even then Lewis, Gren, and Gmelin agree in maintaining, that nothing detrimental is ever to be apprehended from the contamination.

4. But that, Gentlemen, which chiefly induces me to send these remarks to be inserted in your Journal, is a belief, that Mr. Hope's letter seems calculated to divert entirely the attention of those, whom it may influence, from the only certain preventive of hydrophobia. For to this certain preventive, v. g. the timely excision of the bitten part, or to the destruction of that part, in any other way, Mr. Hope's letter does not contain the most faint allusion.

That the importance of this measure may be fully and fairly estimated, your readers must recollect, that it is a law of all those morbid poisons, which act only in a palpable form (e. g. vaccine virus) to excite inflammation at the place of insertion, before the specific disease shows itself in the constitution; and, that a caustic applied to the vaccine pustule, on the seventh day, effectually prevents vaccina; so that whatever may be the interval between the bite of a mad dog and the appearance of hydrophobia, which is constantly fatal, (Dr. Hamilton thinks it happens oftenest between the fourth week and the third month,) the destruction of the bitten part, provided it can be done safely, by excision or by the actual or potential cautery, or the amputation of a limb, provided it be necessary, ought never to be deferred a single moment. Mr. Nourse relates a case, in which the constitutional symptoms did not appear till the nineteenth month; but there is a case on record, in which the excision of the bitten part, performed four days after the bite, did not prevent hydrophobia. I repeat, then, that hydrophobia, like vaccina, is an inoculated disease; that both affect the constitution through the medium of an external and specific inflammation; and that both may be prevented, by preventing this external and specific inflammation. Whether, therefore, Mr. Hope deserve more blame for what he has done, than for what he has neglected to do, let your candid and intelligent Readers determine.

Assuring you, Gentlemen, that I have nothing more at heart than the improvement of medicine, and a desire to see it applied justly and rightly,

*Rochester,* I subscribe myself, &c.

*June* 9, 1805. WALTER VAUGHAN.

